# Evidence of co-circulation of multiple arboviruses transmitted by *Aedes* species based on laboratory syndromic surveillance at a health unit in a slum of the Federal District, Brazil

**DOI:** 10.1186/s13071-021-05110-9

**Published:** 2021-12-19

**Authors:** Paulo Rufalco-Moutinho, Lorena Aparecida Gonçalves de Noronha, Tatyane de Souza Cardoso Quintão, Tayane Ferreira Nobre, Ana Paula Sampaio Cardoso, Daiani Cristina Cilião-Alves, Marco Aurélio Bellocchio Júnior, Mateus de Paula von Glehn, Rodrigo Haddad, Gustavo Adolfo Sierra Romero, Wildo Navegantes de Araújo

**Affiliations:** 1 Center of Tropical Medicine, University of Brasília, Federal District, Brazil; 2 Ceilândia Faculty, University of Brasília, Federal District, Brazil; 3 UNIEURO University Center, Federal District, Brazil; 4 Federal District Department of Health, Federal District, Brazil

**Keywords:** Arboviruses, Dengue, Chikungunya, *Aedes* sp., Syndromic surveillance, Traditional surveillance, Brazil

## Abstract

**Background:**

Vector-borne diseases, especially arboviruses transmitted by *Aedes* sp. mosquitos, should be a health policy priority in Brazil. Despite this urgency, there are significant limitations in the traditional surveillance system, mainly in vulnerable areas. This study aimed to investigate the circulation of dengue (DENV), Zika (ZIKV), and chikungunya viruses (CHIKV) by laboratory syndromic surveillance (LSS) in a slum area of the Federal District of Brazil, comparing the results with traditional surveillance data.

**Methods:**

LSS for acute febrile and/or exanthematous symptoms was developed at a health unit of Cidade Estrutural, in order to identify the circulation of arboviruses transmitted by *Aedes* sp. mosquitos. Between June 2019 and March 2020, 131 valid participants were identified and sera tested by reverse transcription polymerase chain reaction (RT-PCR) for DENV (by serotype), ZIKV, and CHIKV acute infection and by immunoglobulin M enzyme-inked immunosorbent assay (ELISA-IgM) for DENV and CHIKV 15–21 days after symptom onset, when the participant reported no respiratory signs (cough and/or coryza). The results obtained were compared with traditional surveillance data for the study area and period.

**Results:**

At least three DENV-1 (2.3%), four DENV-2 (3%), and one CHIKV (0.7%) cases were confirmed in the laboratory, showing evidence of hyperendemicity even though LSS had not reached the historic peak dengue fever months in the Federal District (April–May). When the results obtained here were compared with traditional surveillance, a significant discrepancy was observed, including underreporting of CHIKV infection.

**Conclusions:**

In addition to the risks posed to the study population, the area investigated with its respective socio-environmental profile may be a potential site for spread of the virus, given the cosmopolitan presence of *Aedes* sp. and human mobility in the Federal District. It is also suggested that traditional epidemiological surveillance may be reporting acute viral infections other than DENV as dengue fever, while underreporting other arboviruses transmitted by *Aedes* sp. mosquitos in the Federal District.

**Graphical Abstract:**

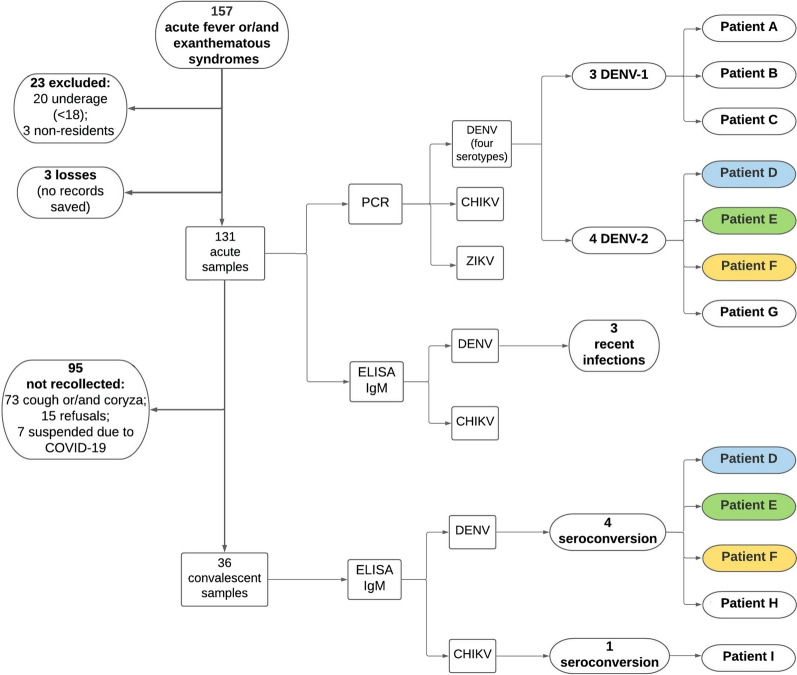

**Supplementary Information:**

The online version contains supplementary material available at 10.1186/s13071-021-05110-9.

## Background

The emergence and re-emergence of neglected infectious diseases (hitherto defined as tropical diseases) require continuous monitoring by the health agencies responsible for surveillance and intervention. This is especially important in Brazil, due to its continental size and large number of biomes under constant environmental disturbance, thereby promoting ecological imbalance. Among the main emerging infectious diseases, arboviruses transmitted by *Aedes* sp. mosquitos warrant special attention in health policies, as observed with the dengue virus (DENV) in the Americas during the first two decades of the twenty-first century. The continent had the largest number of reported cases in the world, with Brazil contributing the highest proportion and exhibiting an endemic–epidemic pattern every 3 to 5 years, in line with the prevalence of the serotype [1]. The country plays a key role in amplification and potentiation of mosquito-borne diseases, as noted by Bedin in 2007 [2], and demonstrated by the *Aedes aegypti*-transmitted Zika virus (ZIKV) epidemic in 2015 which, despite its circulation in other countries, was only confirmed as being associated with congenital malformation syndrome after it emerged in Brazil [3].

With respect to the *Ae. aegypti* vector in Brazil, currently present in all the states of the country since its re-emergence in the 1960s, the transmission of four DENV serotypes stands out: serotypes 1 and 4 in outbreaks during the 1980s, followed by serotype 2 in the 1990s, and serotype 3 in the 2000s [4]. Also, the chikungunya virus (CHIKV) was introduced in the country in 2013 [5], and ZIKV in 2014 [3]. The species is also an urban vector for the yellow fever virus in Brazil, where repeated outbreaks have been observed in recent years, associated with wild vectors (*Haemagogus* sp. and *Sabethes* sp.) in forest areas near urban zones [6]. Finally, the dynamics of other opportunistic arboviruses (Mayaro, o'nyong-nyong, Oropouche, etc.) should be investigated [7, 8] given the vector capacity of *Ae. aegypti* [9].

This current hyperendemic scenario and the complex clinical findings associated with arbovirus infections, such as neuroinvasive disease and Guillain-Barré syndrome, systematically overload the Brazilian health system, affecting the country’s therapeutic and economic capacities [10, 11]. In addition, the congenital malformation syndrome associated with ZIKV infection promotes this agent to the group of STORCH infections (syphilis, toxoplasmosis, rubella, cytomegalovirus, and herpes simplex), thereby requiring prenatal monitoring [12]. The sympatric circulation of different serotypes of DENV and other arboviruses promotes interactions in the pathophysiology and immunological response of the human host, resulting in severe outcomes such as hemorrhagic shocks and death [13].

The consequence of new arboviruses with similar dynamics to those of DENV produces a more complex transmission landscape, resulting in a broad symptomatological spectrum while decreasing the sensitivity and specificity of the clinical-epidemiological criteria for diagnosis, underscoring the need for laboratory confirmation [14]. The high number of asymptomatic and oligosymptomatic infections [15], as well as false-negative diagnoses due to the low sensitivity of widely used methods [16], means that outbreaks related to the arboviruses may occur without timely detection by the epidemiological surveillance system, thereby facilitating their spread and the occurrence of epidemic events [17]. Also considered a complicating factor in controlling mosquito-borne diseases is the systematic dismantling of vector surveillance in recent decades in Brazil, which contrasts with the country’s history of this strategy, such as the eradication of *Ae. aegypti* in the late 1950s [18].

In the present study, laboratory syndromic surveillance (LSS) was conducted for the three arboviruses transmitted by *Aedes* sp. mosquitos in a favela (slum) of the Federal District (FD) of Brazil, for a planned 1-year period, according to the seasonality of mosquito-borne diseases. This study was conducted in the only health unit of the region, assessing patients reporting symptoms associated with acute viral infection by DENV, CHIKV, and ZIKV, following laboratory confirmation by molecular and serological tests. In Brazil, studies involving LSS for arboviruses transmitted by *Aedes* sp. have been carried out in large coastal cities where the population is concentrated, such as the Northeast [10, 14] and Southeast regions [19, 20], but not significantly replicated in the intracontinental portion [21, 22]. The study area has previously been the object of epidemiological investigation based on cross-sectional interviews, reporting 28.6% of the symptoms associated with arbovirus infections in the sample population [23], albeit without laboratory confirmation. The present study aimed to determine the circulation of DENV (by serotype), ZIKV, and CHIKV in the study area; assess the clinical-epidemiological profile of laboratory confirmations; estimate the probable infection site for laboratory confirmations, identifying possible autochthonous transmission; and, given that the study area is geographically isolated and the health unit is the only facility treating the vulnerable population, compare LSS results with traditional epidemiological surveillance data. It is important to underscore that the study was halted in March 2020, before the expected 1-year observation period (June 2020), due to the COVID-19 pandemic.

## Methods

### Study design

Event-based LSS defined by acute arbovirus infection transmitted by *Aedes* sp. was conducted in a health unit, the only facility in the area, and the first destination of individuals seeking medical care. Based on the definition of a probable case in the Manual for Adult and Child Diagnosis and Clinical Management for Dengue Fever [24], acute febrile and/or exanthematous illness was considered the defined event for LSS, thus including a broad-spectrum symptomatology that addressed the symptomatic infections of the three arboviruses investigated here. For DENV, probable case symptomatology is defined as a sharp temperature increase (39–40 °C) accompanied by two or more of the following symptoms: headache, myalgia, arthralgia, and retro-orbital pain, with exanthem present in 50% of symptomatic infections, as well as with anorexia, nausea, vomiting and diarrhea. For CHIKV, symptoms are similar, underscoring the intensity of arthralgia. For ZIKV, symptoms are characterized by absent or low- to moderate-intensity fever, while exanthem occurs more frequently in the first days of the infection.

The sample population consisted of residents in the study area who visited the health unit in the morning (from 8:00 am to 12:00 pm) between Monday and Friday, complaining of acute fever and/or exanthematous illness (the defined event). The primary identification of patients was conducted by family health teams (FHT) [25]. Each FHT is composed of a group of health professionals (doctor, nurse, community health agent) responsible for a geographic area within the respective region served by the health unit, which provides primary care. The health unit where LSS was carried out has 12 FHTs, which are instructed to refer patients presenting with the defined event. After the patient was referred, the following inclusion criteria were established: age 18 years or older; a resident of the study area (at least 4 days a week for at least 3 months before the visit to the health unit); and no contraindications for venipuncture. Patients were excluded if they were homeless or illiterate (given the need to provide written informed consent).

After the patients were referred by the FHT and had agreed to take part in the study, they signed informed consent forms and were given a brief explanation of the study objectives. Blood was collected, which was characterized as the “acute phase” sample, and 15–21 days after symptom onset participants were again contacted for a second blood collection, characterized as the “convalescent phase” sample, provided they did not exhibit cough and/or coryza (flu-like upper respiratory tract syndrome) [26]. The laboratory methodology recommended by the Ministry of Health [24] was applied for molecular and serological confirmation: reverse transcription polymerase chain reaction (RT-PCR) was used for DENV (for serotypes), CHIKV, and ZIKV in acute samples; and immunoglobulin M enzyme-linked immunoassay (ELISA-IgM) for DENV and CHIKV in both acute and convalescent samples. For the ELISA-IgM serological assay, two parameters were established: acute infection when seroconversion occurs, and recent infection when serology is positive in the acute sample [14]. To confirm acute infection, positive RT-PCR and/or seroconversion are considered laboratory evidence. ELISA-IgM was only tested for DENV due to the possible cross-reactivity between DENV and ZIKV (both flaviviruses).

Based on the seasonal pattern of mosquito-transmitted diseases, an initial 1-year observation period was established for the present study, between June 2019 and June 2020. However, given the lack of knowledge regarding SARS-CoV-2 infection at the time, as well as the lack of biosecurity for conducting research at the health unit, it was decided to halt the study in March 2020, after 9 months of observation.

### Study area

**Fig. 1:**
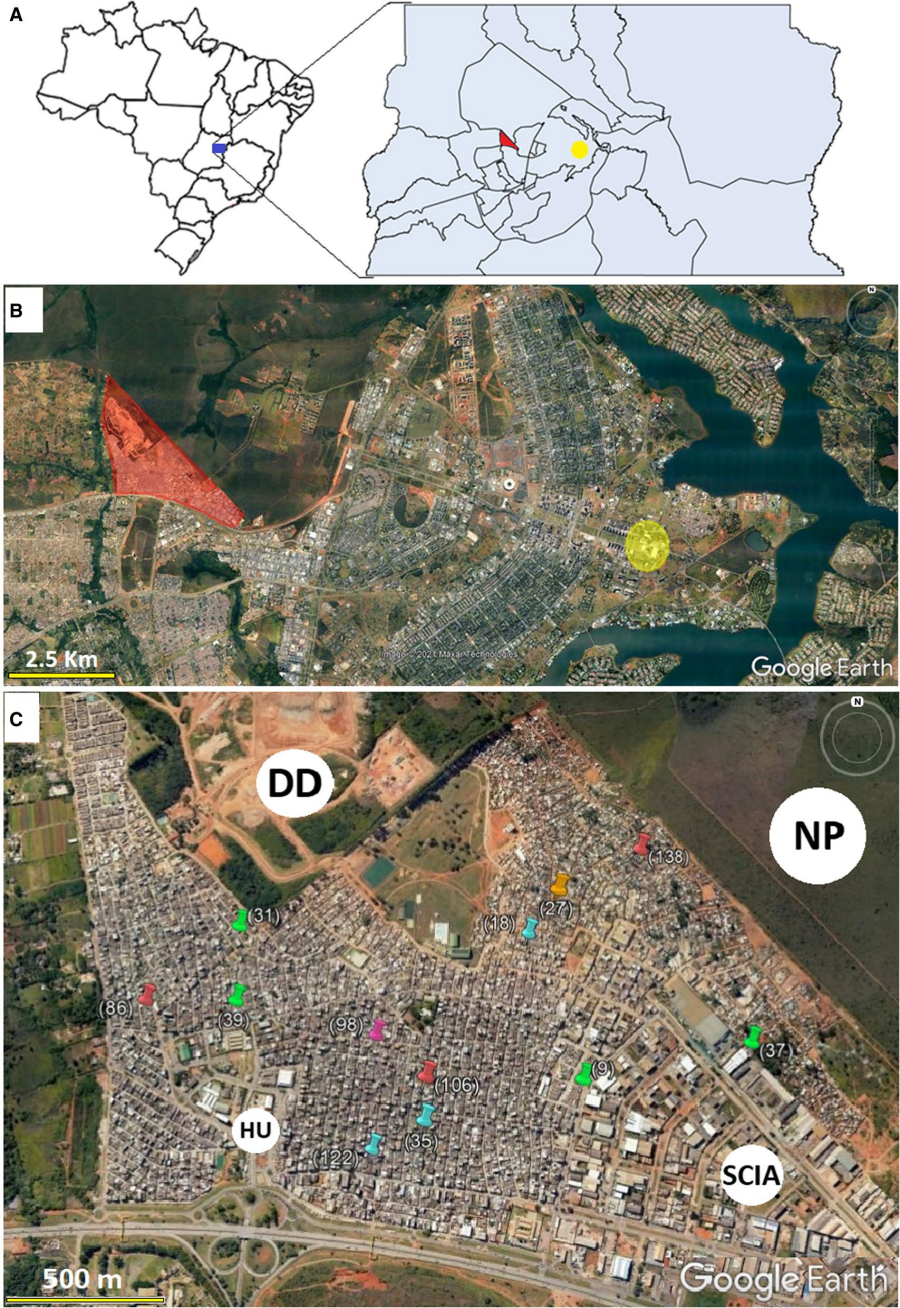
Location of the Federal District in Brazil, highlighting Cidade Estrutural/SCIA in red and Praça dos Três Poderes in yellow (**a**). Satellite image of the pilot project (central region of the Federal District) and surrounding area, highlighting Cidade Estrutural/SCIA in red and Praça dos Três Poderes in yellow (**b**). Satellite image of Cidade Estrutural, highlighting the key locations and homes with positive laboratory confirmations for the arboviruses investigated; *HU* health unit, *DD* deactivated dumping ground, *NP* National Park of Brasilia, *SCIA* AR bordering Cidade Estrutural, commercial and industrial area with a low number of houses (**c**). The homes with positive laboratory confirmations detected by LSS are represented by the following colors: red: DENV-1; green: DENV-2; purple: CHIKV; orange: DENV-IgM (seroconversion); blue: recent DENV infection

LSS was conducted at Health Unit No. 4 (15°4′00.89″S 47°49′49.96″ W) in the Cidade Estrutural administrative region (AR) of the FD (Fig. 1a). Cidade Estrutural is less than 10 km from the political center of Brazil and the executive, legislative, and judiciary branches of government (Fig. 1b). Established as a landfill site in the early 1960s, the area is one of the least developed ARs in the FD, with a vulnerable population that suffers from poverty and a lack of urban planning, infrastructure, and environmental sanitation. A population of approximately 35,730 inhabitants was estimated for 2018, corresponding to 1.23% of the FD population of 2,894,953 [27]. According to the FD Health Department (*Secretária de Saúde do Distrito Federal—SES-DF* in Portuguese), the number of probable cases per 100,000 reported in the Cidade Estrutural in 2016, 2017, 2018, and 2019 was 1132, 475, 92.5, and 1613, respectively, and for the same period in the FD was 583, 141, 79, and 1428.

### Interview and blood sampling

Blood collection at the acute phase was performed using a tube with separator gel (5 ml), after which patients were interviewed to obtain demographic and socioeconomic characteristics (age, sex, ethnicity, address, domestic sanitary conditions), first day of symptoms, clinical characteristics of the event, daily routine in the 15 days prior to symptom onset (school, university, work, or travel), and epidemiological link to an individual (family, neighborhood, work, or schoolmate) with a positive medical diagnosis of DENV. Between 15 and 21 days after symptom onset, participants who did not report cough or coryza were contacted for convalescent blood collection at the conclusion of the second part of the questionnaire (focusing on symptoms reported at the acute phase: cure or persistence).

A standardized questionnaire from the REDCap website, version 7.5.0 (www.project-redcap.org/), was applied using mobile devices (tablets).

### Laboratory preparation

Tubes containing the collected blood were kept at ambient temperature in the health unit laboratory, followed by centrifugation at 1500×*g* for10 min, aliquoting the supernatant serum in triplicate microtubes (usually 0.5 ml in each microtube). Once duly labeled with the respective patient code, the samples were initially kept in the laboratory freezer of the health unit (−20 °C), then taken in thermal containers to the biorepository of the Center for Tropical Medicine, University of Brasilia (UnB), where they were stored (−80 °C) until molecular and serological analyses.

### Viral RNA extraction

Viral RNA extraction of 140 μl of serum from the acute sample was conducted using the QIAamp Mini Kit, according to the manufacturer’s recommended protocol (www.qiagen.com/us/resources), resulting in 60 μl of eluate.

### RT-PCR and arboviral RNA detection

RT-PCR was performed using the ZDC Multiplex Kit from the Institute of Molecular Biology of Paraná with 38 μl of eluate, in accordance with the manufacturer’s recommended protocol (www.ibmp.org.br/en-us/). In this protocol [28], a standardized 96-well microplate is subdivided into four isometric subgroups of 24 wells for the respective target arboviruses: DENV-1/4, DENV-2/3, CHIKV, and ZIKV. Each of these four subgroups on the plate is composed of 23 wells to test the samples (38 µl/4 subgroups = 9.5 µl of sample eluate for each subgroup), and one positive control from the kit itself. For the real-time PCR (qPCR) probes, the ZDC kit uses three triplex reactions and two duplex reactions in the same plate: DENV-1 (HEX), DENV-4 (FAM), and internal control (Quasar 670); DENV-3 (HEX), DENV-2 (FAM), and internal control (Quasar 670); CHIKV (FAM) and internal control (HEX); and ZIKV (FAM) and internal control (HEX) [29].

RT-PCR was amplified using the QuantStudio 5 real-time system (http://thermofisher.com/) at 51 °C for 30 min, followed by 95 °C for 15 min and 40 cycles of 95 °C for 15 s and 60 °C for 60 s. Results were analyzed using the company’s software, and considered positive when the cycle threshold was ≤ 36 for the analyzed virus and ≤ 28 for the internal control.

### Serological testing for anti-DENV IgM

Both acute and convalescent samples were tested for anti-DENV using the Panbio Dengue IgM Capture ELISA kit, according to the manufacturer’s recommended protocol (www.globalpointofcare.abbott). Reading was done on a Kasuaki absorbance reader (450 nm), using Panbio units for cutoff values, following the criteria: < 9 non-reactive; between 9 and 11 inconclusive; > 11 reactive for anti-DENV IgM.

### Serological testing for anti-CHIKV IgM

Both acute and convalescent samples were tested for anti-CHIKV using the EUROIMMUN chikungunya IgM ELISA kit, in line with the manufacturer’s recommended protocol (www.euroimmun.com). Reading was done on a Kasuaki absorbance reader (450 nm), using relative units (RU/ml) for cutoff values, based on the following criteria: < 0.8 RU/ml negative; between 0.8 and 1.1 RU/ml inconclusive; > 1.1 RU/ml positive for anti-CHIKV IgM.

### Data analyses

The number of defined events and laboratory confirmations was illustrated on epidemic curves, organized by epidemiological week (EW) starting with symptom onset. For this, the epidemiological calendar was used for the period of the study (2019 and 2020) [30]: by definition, the first EW ends on the first Saturday of January, as long as it falls at least 4 days into the month. Clinical-epidemiological characteristics were cross-referenced with laboratory confirmations, using frequency tables. Information on routines for the 15 days prior to symptom onset was used to estimate the probable infection site.

In order to compare LSS results with traditional epidemiological surveillance data, probable arbovirus cases reported by the Cidade Estrutural and the FD were extracted from the Ministry of Health (datasus.gov.br) and FD Health Department databases (info.saude.df.gov.br). Epidemiological bulletins from the FD Health Department were consulted to complement this information [31]. The final classification criteria for the probable case (clinical-epidemiological or laboratory) for Cidade Estrutural were obtained, along with the laboratory methods used (rapid NS1 test, IgM serology, PCR, viral isolation) and the dates of the first symptoms and blood collection for the respective laboratory tests established. Epidemic curves of probable DENV and CHIKV cases from traditional surveillance for Cidade Estrutural were organized by EW starting on the day of symptom onset for the study period, discriminating the respective final classification (DENV, CHIKV, discarded, and non-investigated). Probable ZIKV cases were not arranged by EW for the study area due to the unavailability of these reports at the AR level for the FD. In order to observe the statistical differences between traditional surveillance and LSS results, the Wilcoxon test for paired data was conducted. To contextualize the COVID-19 situation in the first quarter of 2020, FD Health Department epidemiological bulletins from the Center of Emergency Operations for COVID-19, established in February of that year, were consulted [32].

Microsoft Excel was used to manage the datasets and create tables and graphs; Stata 14.0 software was used for statistical analyses, and Google Earth for satellite images. The GPS points of the dwellings were collected using a Garmin eTrex 10 handheld navigator.

## Results

The study period was between the third week of June (EW 25) 2019 and third week of March (EW 12) 2020. A total of 157 individuals who presented with the defined event were referred by the FHT, 134 of whom were considered eligible for the study. The information on three of these was not saved electronically. Ultimately, 131 defined events were identified by the study. For the second blood collection (convalescent phase), the samples of 36 individuals were collected, given that 73 defined events exhibited cough and/or coryza, 15 individuals refused to participate, and seven were not approached due to the early termination of the study. Demographic and socio-environmental characteristics are presented in Table 1. The LSS population samples consisted of adults around 30 years of age, with approximately 10 years of schooling (0 never attended; 16 had university graduate level), most of whom were non-white (89%) and were women (62.6%).

**Table 1:** Demographic and socio-environmental characteristics of individuals identified by LSS at a Cidade Estrutural health unit, between June 2019 and March 2020

Characteristics	Frequency or mean *N* = 131	% or SD
Age (years)	32.2	12.8
Sex (female)	81	62
Ethnicity (non-white)	118	90
Years of schooling	9.2	4.5
No health insurance	124	94.6
Sewage collection at home	106	80.9
Indoor plumbing	106	80.9
Water rationing in the past 3 months	52	39.7
Stores water by other methods	45	34.3
Trash collection at home	114	87

*SD* standard deviation

For positive laboratory confirmation, LSS identified three individuals with acute DENV-2 infection, testing positive for RT-PCR and negative for ELISA-IgM at the acute phase, followed by seroconversion at the convalescent phase; one individual with acute DENV-2 infection detected only by RT-PCR, whose serology in both the acute and convalescent samples was negative; three individuals with acute DENV-1 infection, positive for RT-PCR and negative for ELISA-IgM at the acute phase but with no serology performed at the convalescent phase (one of the participants refused the second collection; for the other two, LSS had already been terminated); one with acute DENV infection, detected only by seroconversion, defined DENV-IgM; one with acute CHIKV infection, detected only by seroconversion; and three recent DENV infections, as per a positive ELISA-IgM result from the acute sample, and negative RT-PCR. The homes that exhibited positive laboratory confirmation were georeferenced (Fig. 1c).

**Fig. 2:**
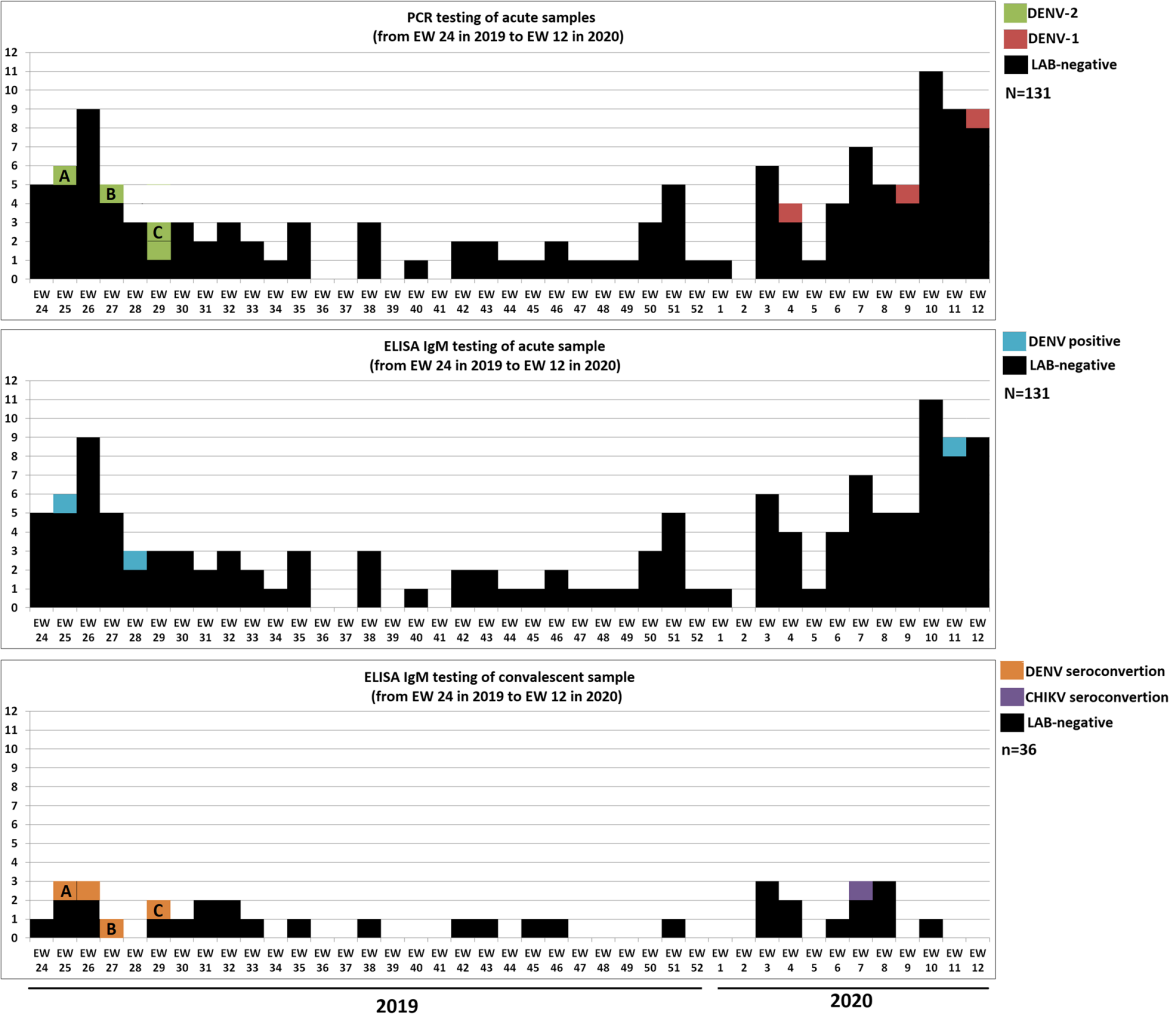
Epidemic curves for laboratory confirmations for LSS symptom onset data between EW 24 of 2019 and EW 12 of 2020. RT-PCR testing for acute samples (131); ELISA-IgM testing for acute samples (131); and ELISA-IgM testing for convalescent samples (36). **a**, **b** and **c** on the RT-PCR and Elisa-IgM convalescent samples of the same individuals laboratory-confirmed for acute DENV-2 infection

Figure 2 shows three epidemic curves during the study period, adjusted according to collection time and the respective laboratory methodology used. The four confirmations of DENV-2 and one of DENV-IgM were observed between EW 25 and EW 29 (June–July) of 2019, while the three DENV-1 and one CHIKV confirmations were found between EW 4 and EW 12 (January–March) of 2020. No positive laboratory confirmation was observed in the period between these two clusters (second half of 2019). Despite the significant increase in defined events in the last EWs before the end of the study, only one positive laboratory confirmation was identified in the acute samples.

The clinical-epidemiological characteristics of the 132 defined events and respective laboratory confirmations are shown in Table 2. Nearly all of the five most common symptoms associated with acute viral infections (headache, fever, myalgia, arthralgia, and retro-orbital pain) were observed in positive laboratory confirmations, except for one DENV-1 without arthralgia and two DENV-2 without arthralgia or retro-orbital pain. Cough and coryza were observed in one DENV-1 and one DENV-2 case, respectively. Exanthem was present in DENV IgM (identified in June 2019) and two DENV-1 confirmations (identified in January–March 2020). CHIKV stands out for the presence of sore throat. Although alarming signs were reported (difficulty breathing, bleeding), no severe dengue fever was found.

**Table 2:** Clinical-epidemiological characteristics and laboratory diagnosis of 131 defined events identified at the Cidade Estrutural health unit

Clinical signs	Laboratory results					
	Total tested *N* = 131	DENV-1 (*n* = 3)	DENV-2 (*n* = 4)	DENV-IgM (*n* = 1)	CHIKV (*n* = 1)	Negative (*n* = 122)
Headache	128	3	4	1	1	119
Fever	125	3	4	1	1	116
Myalgia	126	3	4	1	1	117
Arthralgia	103	2	3	1	1	96
Retro-orbital pain	102	3	3	1	1	94
Nausea	92	1	3	0	1	87
Difficulty swallowing	77	2	2	0	1	72
Shortness of breath	55	0	2	0	1	53
Conjunctivitis	55	2	1	1	0	51
Diarrhea	44	1	2	0	0	41
Exanthem	19	2	0	1	0	16
Vomiting	35	0	2	0	0	33
Itching	33	1	0	1	0	31
Cough	66	1	0	0	0	65
Coryza	62	0	1	0	0	61
Sore throat	62	0	0	0	1	61
Bleeding^a^	18	2	0	0	0	16
Oral mucosal lesion	15	1	0	0	0	14

^a^Nose, mouth, or feces

The main characteristics related to the daily routines and possible exposure for laboratory confirmation by LSS are presented in Additional file 1: Table S1. Individuals from samples 39 (DENV-2), 98 (CHIKV), and 106 (DENV-1) were not exposed outside their homes, and these cases can be plausibly characterized as household transmission. For individual 27 (DENV-IgM), Cidade Estrutural can also be considered the probable infection site (residence or workplace). The workplace of individual 37 (DENV-2) was in the neighboring AR (SCIA, see Fig. 2c), but since this individual worked only at night, when *Ae. aegypti* activity declines significantly [9], household transmission is also suggested in this instance.

**Fig. 3:**
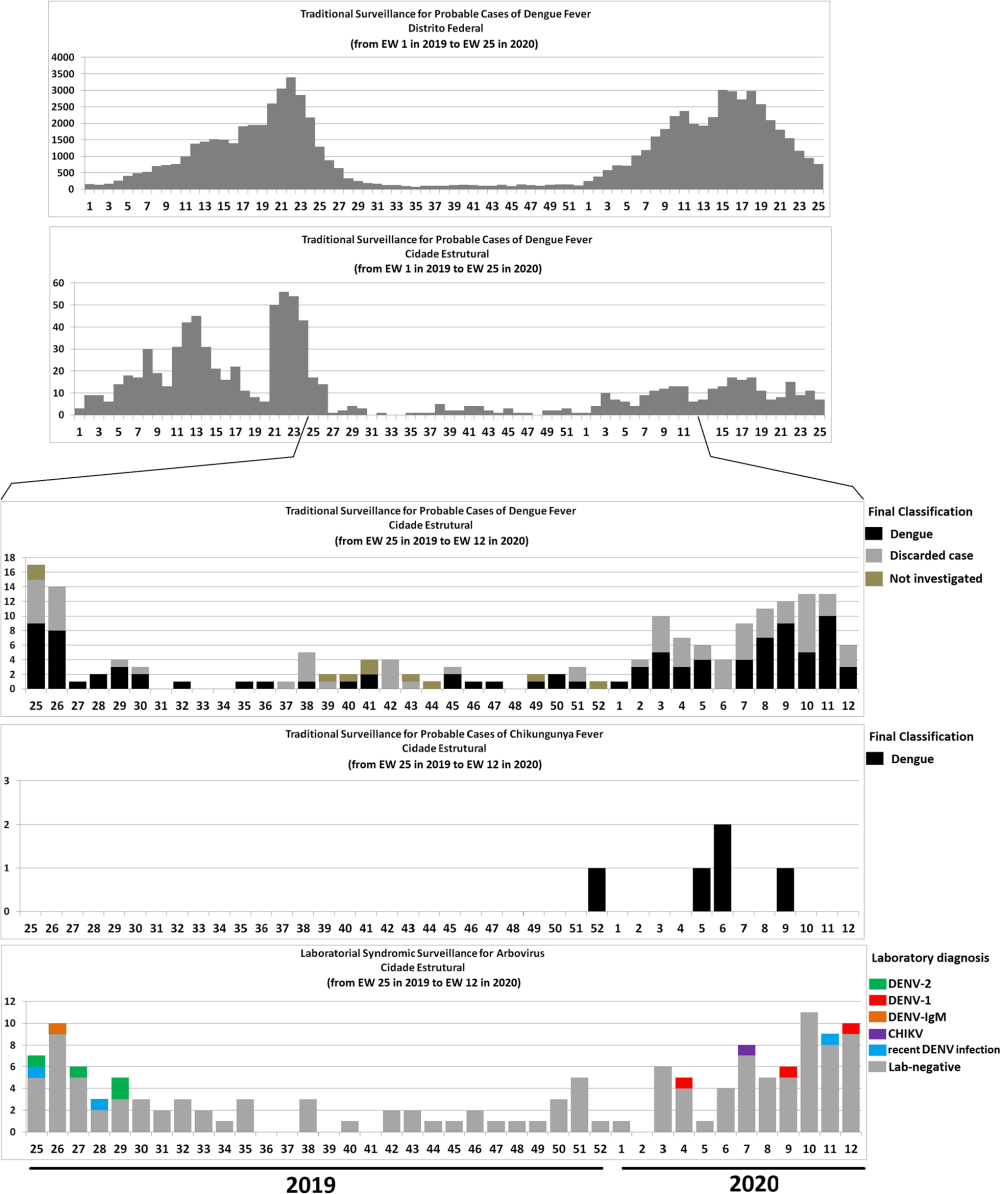
From top to bottom, epidemic curves of symptom onset for traditional surveillance of probable DENV cases in the Federal District between EW 1 of 2019 and EW 25 of 2020; traditional surveillance of probable DENV cases in Cidade Estrutural between EW 1 of 2019 and EW 25 of 2020; traditional surveillance of probable DENV cases and final classification for Cidade Estrutural between EW 25 of 2019 and EW 12 of 2020; traditional surveillance of probable CHIKV cases and final classification for Cidade Estrutural between EW 25 of 2019 and EW 12 of 2020; and LSS results between EW 25 of 2019 and EW 12 of 2020

Figure 3 shows five epidemic curves. The first two, from top to bottom, correspond to traditional surveillance reports of probable DENV cases for the FD and Cidade Estrutural, respectively, between EW 1 of 2019 and EW 25 of 2020. The three remaining Cidade Estrutural curves during the study (EW 25 of 2019 to EW 12 of 2020) are for probable DENV cases reported (characterized according to the final classification), probable CHIKV cases reported (characterized according to the final classification), and LSS results, respectively. The traditional surveillance data for the FD show that significant peaks of probable DENV cases occurred during the first half of both years (2019 and 2020), but only in 2019 was a peak observed in Cidade Estrutural. There is a pandemic bias for 2020, contextualized in the Discussion section.

Figure 3 shows the comparisons between traditional surveillance reports (174 probable DENV cases during the study period, 94 classified as DENV, 70 discarded, and 10 not investigated; and five probable CHIKV cases, all classified as DENV); and LSS results (126 defined events, with nine laboratory confirmations, including CHIKV), revealing differences between magnitudes and distributions. In regard to the EWs for LSS confirmations, the first cluster exhibited five confirmations of acute infection (four DENV-2 and one DENV-IgM) between EW 25 and EW 29 of 2019, the same period during which traditional surveillance identified 23 DENV cases; and the second cluster, four confirmations of acute infection (three DENV-1 and one CHIKV) between EW 4 and 12 of 2020, the same period in which traditional surveillance reported 45 DENV cases. Information on traditional surveillance also indicates DENV cases being reported during the second half of 2019, a pattern not observed by LSS. Although the probable CHIKV cases of traditional surveillance are temporally close to LSS confirmation, they were classified as DENV.

With respect to statistical analyses between traditional surveillance data and LSS results, considering a 95% confidence interval (CI) and the null hypothesis that the magnitudes of EW-paired values are equal, differences were observed for all the comparisons performed: between probable DENV cases and defined events (*P* = 0.0346); between DENV cases and defined events (*P* = 0.0238); and between DENV cases and laboratory confirmations (*P* = 0.000). In terms of the traditional surveillance criteria used to confirm probable DENV cases for Cidade Estrutural in 2019, 571 of the 652 probable cases reported were thoroughly investigated, 216 using clinical-epidemiological criteria and 355 laboratory methods. However, these numbers may be inconsistent, since 360 rapid tests were conducted for NS1, in addition to 167 serological IgM, five PCR, and one viral isolation test. For 2020, 310 of the 312 probable DENV cases reported were thoroughly investigated: 44 using clinical-epidemiological criteria and 266 laboratory methods (231 by rapid NS1 testing, 157 by IgM serology, 24 by PCR, and 1 by viral isolation).

## Discussion

Sentinel units are an important strategy for data collection in health surveillance, and are selected based on the probable occurrence of outcomes of interest, with the area receiving intensive monitoring from a specialized team [33]. For epidemiological surveillance, hospitals and outpatient facilities are important sentinel units for diseases and complications related to human health, since these establishments are the entry points to the health system. With regard to infectious diseases, the natural history and transmission pathway should be parameters for selecting sentinel units. Vulnerable areas such as Brazilian favelas are hot spots for mosquito-borne diseases, according to the association between *Aedes* sp. biology and regions without environmental sanitation [14]. The health unit in Cidade Estrutural is an important satellite for epidemiological surveillance in the FD for investigating vulnerable areas, a situation that underscores the extreme inequality that still exists in Brazil. Given the advantages of well-incorporated and consolidated syndromic surveillance [34], producing accurate real-time results for health authorities [35], opportunities for intervention and control are created before pathogen dissemination. For mosquito-borne diseases, entomological surveillance should also be incorporated to block the transmission chains. These different approaches, which naturally complement each other, represent integrated surveillance [36], an important component in reducing the number of vector-borne diseases, and may even culminate in eradication [37, 38].

The study found evidence of two DENV serotypes and CHIKV in circulation, with high plausibility of at least three individuals having been infected in their homes (positive for DENV-1, DENV-2, and CHIKV). Cidade Estrutural is hyperendemic for arboviruses transmitted by *Aedes* sp. mosquitos, with the population exposed to the risk of secondary infection, co-infection, and severe clinical outcomes in areas where this “arboviral soup” occurs [13], despite cross-immunity being observed among flavivirus infections [39, 40]. The site is a potential area for the spread of these arboviruses to other ARs, given the cosmopolitan presence of *Ae. aegypti* throughout the FD and the human mobility that occurs in the region.

The possible low number of laboratory confirmations by LSS for the population sample may be due to the inclusion of nonspecific symptoms in the clinical spectrum of the defined event, including upper respiratory tract symptoms (cough 66; coryza: 62; both 53) which are not associated with the definition of probable cases for arboviruses transmitted by *Aedes* sp. [24]. This option was adopted to maximize sampling for detection by laboratory methodologies, and to observe the clinical profile of laboratory confirmations. However, when compared to other LSS studies on arboviruses transmitted by *Aedes* sp. conducted in health units in the country, the number of positive DENV cases observed here (6.1%) was higher than that reported by Silva et al. (3.4%) [14], Ferreira et al. (0.4%) [10], Carvalho et al. (0.8%) [19], and Vieira et al. (*n* = 0) [22]. For the other two arboviruses, these studies obtained higher results. In addition to the particularities of each investigation and the study population, this discrepancy depends on whether outbreaks and epidemics occur while LSS is being carried out, as occurred in the study by Silva et al. [14] during the 2015 ZIKV epidemic in Salvador, Bahia state.

Laboratory confirmations exhibited the five most common symptoms involving acute viral infections, confirming the low specificity for diagnosing arbovirus infection when not supported by laboratory analysis [14]. Cough and coryza were observed in one DENV-1 and one DENV-2 confirmation, respectively. This aspect of acute viral syndromes is used to discriminate airborne viruses from others (such as arboviruses) [26]. However, although uncommon, upper respiratory tract symptoms may be associated with acute arboviral infections transmitted by *Aedes* sp., including cough, coryza, and nasal congestion [41]. It is important to underscore that DENV has been isolated in secretion samples collected from the upper respiratory tract, raising the hypothesis of possible transmission via aerosol or close contact, as observed in the replication of this virus in culture media of airway epithelial cells. However, evidence is still insufficient to confirm this infection pathway [42].

Traditional surveillance reporting shows that the difference between probable DENV cases in the FD and Cidade Estrutural indicates that the latter did not contribute to the total in 2020, only 2019. The COVID-19 pandemic represents a potential bias for probable case reports for 2020, starting in March (Additional file 2: Figure S1). Comparisons between traditional surveillance reports and LSS results show important discrepancies, given that it is an isolated area with a vulnerable population dependent on the health unit. Moreover, because traditional surveillance such as LSS had the same source of patients in the health unit, some similarity was expected between the values of the two surveillance methods. However, there were a number of limitations, including the communication between LSS and a number of FHTs, the fact that LSS was performed only in the morning (8:00 am–12:00 pm), the more encompassing symptomatological spectrum of the defined event of LSS when compared with the definition of probable arbovirus cases, the restriction to only those individuals aged 18 years or older, and possible reporting sources other than the health unit, which may explain the difference in the numbers between the two surveillance methods.

The obvious contrast between DENV cases reported by traditional surveillance and LSS confirmations indicates that other acute viral infections may be reported as DENV. The absence of confirmed reports of CHIKV, with probable cases of this virus classified as DENV, suggests underreporting. With respect to laboratory criteria sensitivity, it is known that the rapid tests routinely used for point-of-care testing in outpatient facilities may exhibit low sensitivity when compared to those obtained in the laboratory [43]. For IgM serology, the measures of central tendency for the set of values obtained by the difference between the day of symptom onset and the blood collection day for 2019 (*n* = 167) show a mean of 10.4 days, standard deviation (SD) of 6.4 days, median of 3 days, and mode of 2 days; and for 2020 (*n* = 157), a mean of 4.3 days, SD of 6.6 days, median of 3 days, and mode of 3 days. These indicators reveal that a large number of samples were collected within 1 week of symptom onset, suggesting that recent infections could be classified as acute, according to IgM circulation time in the bloodstream (3 months or more after the end of the infection) [24]. Depending on the seasonality of mosquito-borne diseases, longer transmissibility times may maximize this bias. PCR and viral isolation were less frequently used than the rapid NS1 tests and IgM serology.

Given the limitations of the clinical-epidemiological criteria, and the dependence on an effective flow for laboratory confirmation of infectious diseases, it can be suggested that acute infections other than DENV are reported as DENV for the study area, while other arboviruses are underreported. Several areas of Brazil have vulnerable populations that are dependent on the National Health System (SUS, in Portuguese), which has undergone a structural and financial decline in recent years [44]. The worst scenario has been observed during the COVID-19 pandemic, considering the areas with active arbovirus transmission [45]. The endemic circulation of SARS-CoV-2 in the Brazilian population is a further limiting factor to the accurate reporting of acute viral syndromes when not confirmed by laboratory criteria.

A number of limitations were observed. First, communication with different FHTs produced heterogeneous identification of defined events during the study period, due to limitations of the teams themselves. Second, the LSS could not be maintained during the daily operation of the health unit (8:00 am–6:00 pm), and not collecting the convalescent samples of some patients contributed to reducing the sampling effort for laboratory analysis. Third, the COVID-19 pandemic precluded the completion of the 1-year observational period and delayed the laboratory processing of biological samples, since resources and personnel were redirected to combat the new coronavirus. The pandemic can also be considered a potential bias for the FHT in identifying defined events, and for traditional surveillance data in 2020. However, according to Additional file 2: Figure S1 and the Ministry of Health’s recognition of the existence of community transmission of SARS-CoV-2 on March 20 [32], during the pre-pandemic context LSS identified DENV-1 (EW 4) and CHIKV (EW 7).

The LSS results raise questions regarding the time elapsed between laboratory confirmation of DENV-2 and DENV-1 during the second half of 2019, and the absence of confirmation between these two clusters. Is this transmission sustained at the local level in the study area during low *Aedes* sp. density, depending on the seasonality of mosquito-borne diseases? In addition, does any type of ecological substitution of DENV serotypes occur during this seasonality? Thus, to answer these questions, active surveillance should be performed (also focusing on asymptomatic individuals), along with entomological surveillance and analyses of mosquito infectivity.

## Conclusion

Despite the limitations, LSS identified autochthonous household transmission for two different DENV serotypes and for CHIKV. When these results are compared with those of traditional surveillance, there is a significant discrepancy, suggesting that other acute viral syndromes are classified as DENV, and that other arboviruses transmitted by *Aedes* sp. are underreported. Given the high number of negative laboratory results observed by LSS, acute syndromes caused by other viruses can be speculated, including airborne viruses, as demonstrated by upper respiratory tract symptoms (flu-like syndrome), which circulated in the FD before the COVID-19 pandemic (influenza virus, adenovirus, rhinovirus, earlier coronaviruses, etc.), indicating the importance of continuous LSS for surveillance strategies. Integrated surveillance methods (traditional, syndromic, laboratory-based, genomic, entomological), in addition to hybrid systems, new data science methodologies, and the feeding of predictive models [46], are essential instruments in predicting the emergence of new and re-emergence of earlier infectious agents in this brave new world.

## Supplementary Information

**Additional file 1: Table S1.** Infection characteristics, blood collection dates, daily routines and possible exposures of LSS acute cases at the Cidade Estrutural health unit.

**Additional file 2: Figure S1.** Epidemic curve by day of symptom onset of confirmed COVID-19 cases according to evolution in the Federal District to August 27, 2020, highlighting the LSS final period (between EW 9 and EW 12).

## Data Availability

The datasets used and/or analyzed during the current study are available from the corresponding author on reasonable request.

## Abbreviations

STORCH: Syphilis, toxoplasmosis, rubella, cytomegalovirus, and herpes simplex
LSS: Laboratory syndromic surveillance
DENV: Dengue virus
ZIKV: Zika virus
CHIKV: Chikungunya virus
FHT: Family health team
AR: Administrative region of the Federal District, Brazil
FD: Federal District
EW: Epidemiologic week
