# Supplementary material for: Evidence of co-circulation of multiple arboviruses transmitted by Aedes species based on laboratory syndromic surveillance at a health unit in a slum of the Federal District, Brazil
Source: Parasit Vectors. 2021 Dec 19;14:610. doi: 10.1186/s13071-021-05110-9 (PMC8684590; doi:10.1186/s13071-021-05110-9)
Supplement: Supplementary file 1 — Additional file 1: Table S1. Infection characteristics, blood collection dates, daily routines and possible exposures of LSS acute cases at the Cidade Estrutural health unit. [file 13071_2021_5110_MOESM1_ESM.docx]

**Additional file 1:** Table S1. Infection characteristics, blood collection dates, daily routines and possible exposures to lab-confirmed arbovirus cases at the Cidade Estrutural health unit

| **Samples** | **Lab-diagnoses** | **Day of symptom onset**  **(dd/mm/yy)** | **Acute**  **blood**  **collection**  **(dd/mm/yy)** | **Convalescent**  **blood**  **collection**  **(dd/mm/yy)** | **Daily**  **routine** | **Travel**  **In last 15 days** | **Exposure**  **In last 15 days** | **Acute**  **Symptoms** |
| --- | --- | --- | --- | --- | --- | --- | --- | --- |
| 9 | DENV-2 | 16/06/19 | 18/06/19 | 01/07/19 | Unemployed/  not attending school or university | Rural zone  of other AR | No | headache; fever; myalgia; arthralgia; retro-orbital pain; diarrhea |
| 27 | DENV-IgM | 29/06/19 | 02/07/19 | 14/07/19 | Works during the day in study area/  not attending school or university | No | Co-worker  with dengue fever | headache; fever; myalgia; arthralgia; retro-orbital pain; conjunctivitis; itching; exanthem |
| 31 | DENV-2 | 06/07/19 | 08/07/19 | 21/07/19 | Works during the day in the FD/  not attending school or university | Rural zone  of Goias state | Co-worker and neighbor  with dengue fever | headache; fever; myalgia; arthralgia; nausea; shortness of breath; coryza; difficulty swallowing |
| 37 | DENV-2 | 17/07/19 | 19/07/19 | 01/08/19 | Works at nightin the study area/  not attending school or university | No | Co-worker and neighbor  with dengue fever | headache; fever; myalgia; arthralgia; retroorbital pain; nausea; vomit; diarrhea; shortness of breath; conjunctivitis; difficulty swallowing |
| 39 | DENV-2 | 18/07/19 | 23/07/19 | 02/08/19 | Works at home/  not attending school or university | No | Family member in the same home  with dengue fever | headache; fever; myalgia; nausea; retro-orbital pain; vomiting |
| 86 | DENV-1 | 21/01/20 | 23/01/20 | - | Works during the day in another AR/  not attending school or university | No | Neighbor  with dengue fever | headache; fever; myalgia; arthralgia; retro-orbital pain; nausea; cough; conjunctivitis; difficulty swallowing; itching; exanthem |
| 98 | CHIKV | 11/02/20 | 14/02/20 | 26/02/20 | Unemployed/  not attending school or university | No | No | headache; fever; myalgia; arthralgia; retro-orbital pain; nausea; shortness of breathe; sore throat; difficulty swallowing |
| 106 | DENV-1 | 24/02/20 | 27/02/20 | - | Works during the day in another AR /  not attending school or university | No | No | headache; fever; myalgia; arthralgia; retro-orbital pain; diarrhea; conjunctivitis; difficulty swallowing; oral strategyl lesion; exanthem; bleeding (mouth, while brushing teeth) |
| 138 | DENV-1 | 18/03/20 | 20/03/20 | - | Attending school or university in the daytime in another AR | No | No | headache; fever; myalgia; diarrhea; retro-orbital pain; bleeding (feces) |

FD: Federal District; AR: Administrative Region
