# Supplementary figures and images for: Evidence of co-circulation of multiple arboviruses transmitted by Aedes species based on laboratory syndromic surveillance at a health unit in a slum of the Federal District, Brazil
Source: Parasit Vectors. 2021 Dec 19;14:610. doi: 10.1186/s13071-021-05110-9 (PMC8684590; doi:10.1186/s13071-021-05110-9)

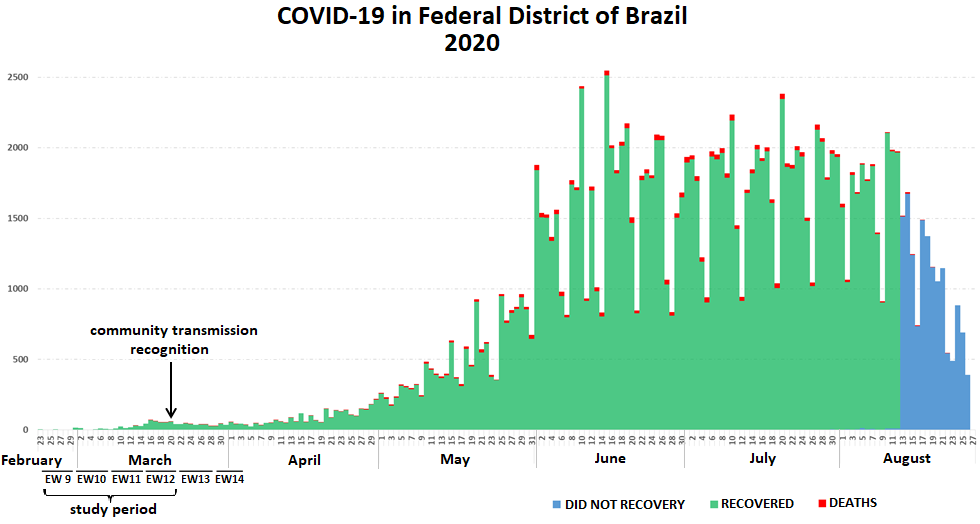

Supplement: Supplementary file 2 — Additional file 2: Figure S1. Epidemic curve by day of symptom onset of confirmed COVID-19 cases according to evolution in the Federal District to August 27, 2020, highlighting the LSS final period (between EW 9 and EW 12). [file 13071_2021_5110_MOESM2_ESM.png]
